# Metastatic Lung Adenocarcinoma Presenting with Hypereosinophilia

**DOI:** 10.7759/cureus.2866

**Published:** 2018-06-22

**Authors:** Omar Abughanimeh, Mohammad Tahboub, Mouhanna Abu Ghanimeh

**Affiliations:** 1 Internal Medicine, University of Missouri Kansas City School of Medicine, Kansas City, USA; 2 Internal Medicine, University of Missouri at Kansas City, Kansas City, USA; 3 Internal Medicine/Gastroenterology, Henry Ford Health System, Detroit, USA

**Keywords:** eosinophilia, hypereosinophilia, metastatic lung adenocarcinoma

## Abstract

Lung cancer is one of the most common malignancies in both male and female patients. It is classified into small cell lung cancers and non-small cell lung cancers. Lung adenocarcinoma is a subtype of non-small cell lung cancer and accounts for the highest prevalence of lung cancer. Eosinophils are white blood cells (WBCs) that originate from the granulocytic lineage. Hypereosinophilia is a rare condition characterized by an absolute eosinophil count (AEC) of more than 1500 cells/µL. This is different from eosinophilia, which is defined as an absolute eosinophil count of more than 500 cells/µL. Hypereosinophilia is associated with several conditions, including allergic disorders, helminth infections, rheumatologic disorders, and hematologic malignancies. Paraneoplastic eosinophilia is a rare finding in solid malignancies. Herein, we report the case of a 55-year-old male who presented with shortness of breath and chest pain and whose workup showed metastatic lung adenocarcinoma associated with hypereosinophilia in the absence of a primary bone marrow disorder.

## Introduction

Lung cancers are very common and account for more than 10% of all malignancies. Lung cancer is considered to be the leading cause of malignancy-related deaths in both males and females [1]. Lung cancer is classified as either small cell lung cancer (SCLC) or non-small cell lung cancer (NSCLC); both are associated with paraneoplastic diseases. Eosinophils are white blood cells (WBCs) derived from hematopoietic stem cells that differentiate into a myeloid lineage. Eosinophilia is defined as an absolute eosinophil count (AEC) of more than 500 cells/µL. Hypereosinophilia is a more severe condition and is defined as having an AEC greater than 1500 cells/µL, without organ damage [2-4]. There are many causes of eosinophilia which include parasitic infections, allergic diseases, rheumatologic diseases, and neoplasms. Eosinophilia in malignancy is usually caused by hematologic malignancies. However, solid tumors can cause eosinophilia in some cases [5]. The incidence of eosinophilia in malignant tumors is estimated to be approximately 1% [6]. Eosinophilia presentation can be silent and life-threatening. The cause of eosinophilia in solid malignancies is thought to be related to bone marrow stimulation through cytokines such as interleukin-5 (IL-5) [5]. There is no specific treatment for paraneoplastic eosinophilia other than treating the underlying malignancy [4].

## Case presentation

**Figure 1:**
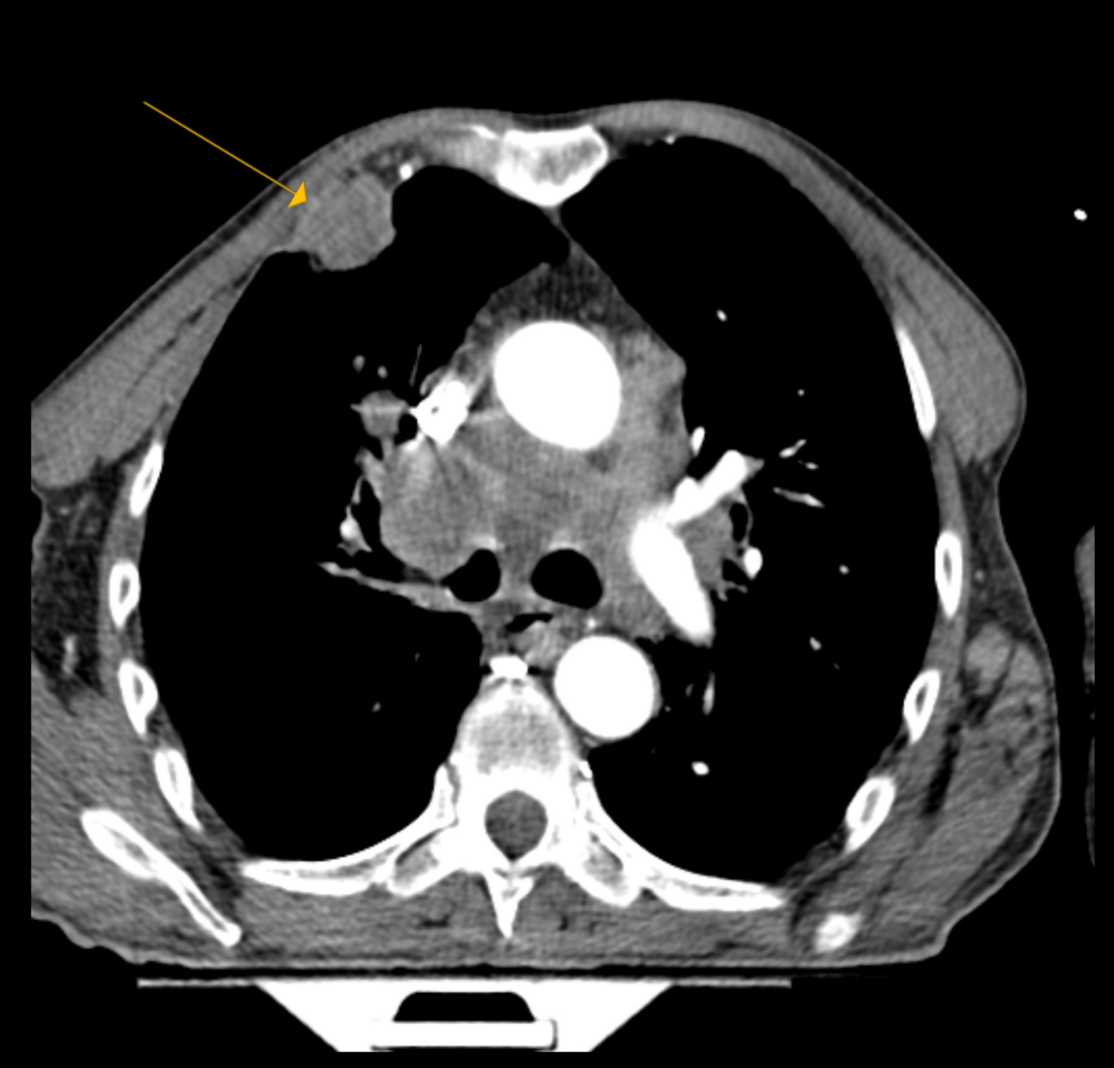
A CT pulmonary angiogram showing a 3.6-cm speculated mass within the anterior right upper lobe CT: computed tomography

A 55-year-old Caucasian male, with a past medical history significant for tobacco abuse (41 pack-years), presented with shortness of breath accompanied by chest and back pain for two months. Blood workup showed a WBC count of 68,400 cells/µL, with an AEC of 27,360 cells/µL. A computed tomography (CT) pulmonary angiogram was performed, as he was hypoxic, and revealed a 3.6-cm speculated mass within the anterior right upper lobe, partially invading the anterior chest wall. It also revealed mediastinal and hilar adenopathy, an extensive osseous lesion (including compression fracture at T7), and a small pericardial effusion (Figure 1). A CT of the abdomen and pelvis with contrast was performed and revealed a diffuse metastatic disease involving the liver, adrenal glands, spleen, and the bones. Magnetic resonance imaging (MRI) of the thoracic spine did not reveal spinal cord compression, but it did show the compression fracture at T7 and multilevel thoracic spondylosis. An MRI of the brain revealed a 5-mm lesion in the left occipital lobe, without edema or mass effect.  

The hematology-oncology team was consulted for an evaluation of the metastatic disease and the eosinophilia. A core needle biopsy was obtained from a liver lesion and the result came back as poorly differentiated adenocarcinoma of the lung (cytokeratin 7, TTF1, and napsin-A were positive, while cytokeratin 2 and CDX2 were negative). Given his functional status, the decision was made to hold on systemic therapy and start on palliative radiation to the spine for pain control. The plan was to complete radiation sessions and then evaluate his functional status before starting systemic therapy.

**Figure 2:**
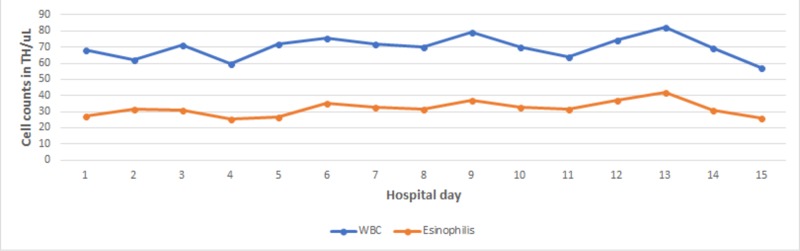
WBCs and eosinophils trend during admission

He continued to have a high WBC count during the admission (Figure 2). Therefore, a bone marrow biopsy was performed to rule out a hematologic malignancy and it revealed metastatic adenocarcinoma of the lung with no evidence of a myeloproliferative disorder. The flow cytometry from the bone marrow showed a CD5-positive clonal B-cell population, which was similar to the blood flow cytometry. Blood tests, including tests for Janus kinase 2 (JAK-2), calreticulin (CALR), MPL, BCR-ABL, and platelet-derived growth factor receptor (PDGFRA), were negative. The blood smear showed microcytic anemia with leukocytosis with absolute neutrophilia and eosinophilia. The serum immunoglobin E (IgE) was high at 377 IU/ml, and the tryptase level was low at 1.8 µg/L. Given these findings, his eosinophilia was related to a paraneoplastic process rather than a primary bone marrow disease.

During the following days, the patient completed 13 sessions of radiation without improvement in his functional status, pain, or breathing. The case was discussed with the patient and his family; he decided that he would go with comfort measures, so he was discharged to the hospice facility.

## Discussion

Lung cancers are one of the most common solid organ malignancies. They account for 10% of all malignancies and are considered to be the leading cause of death in both genders [1]. Lung cancers have been associated with different paraneoplastic syndromes. However, paraneoplastic eosinophilia is rarely reported in the English literature.

Eosinophils are WBCs that are derived from the hematopoietic stem cells that differentiate into the myeloid lineage. In the blood smear, these cells display a bilobed nucleus with an abundant cytoplasm, which is filled with pinkish granules upon eosin stain [2-3]. Eosinophilia is defined by an AEC of more than 500 cells/µL. A more severe form, called hypereosinophilia, is defined as an AEC greater than 1500 cells/µL, without organ damage [4]. There are many causes of eosinophilia, which include parasitic infections, allergic diseases, rheumatologic diseases, and hematologic malignancies. There are some reported cases of eosinophilia that were associated with solid organ malignancies such as thyroid, gastrointestinal, and breast cancer [7]. Table 1 summarizes the reported cases of paraneoplastic eosinophilia secondary to lung cancer in the english literature.

**Table 1:** Reported cases of paraneoplastic eosinophilia secondary to lung cancer in the English literature

Case	Age/Gender	Lung cancer	Max absolute eosinophils count
Slungaard et al. [3]	71 years/male	Large cell carcinoma	6250 cell / µL
Nqwata et al. [4]	52years/male	Adenocarcinoma	82330 cell / µL
Lo CH et al. [6]	82 years/male	Adenocarcinoma	32748 cell / µL
El-Osta et al. [7]	53 years/ male	Large cell carcinoma	14560 cell / µL
Matsumoto et al. [8]	55 years/ male	Squamous cell carcinoma	897 cell / µL
Pandit et al. [9]	72 years/male	Large cell carcinoma	89110 cell / µL
Sawyers et al. [10]	65 years/male	Squamous cell carcinoma	30770 cell / µL
Sawyers et al. [10]	68 years/male	Adenocarcinoma	11439 cell / µL
Motilalet al. [11]	35 years/male	Adenocarcinoma	11300 cell / µL
Machaczka et al. [12]	59 years/male	Adenocarcinoma	3750 cell / µL
Kodama et al. [13]	52 years/ male	Large cell carcinoma	1128 cell / µL
Henry et al. [14]	72 years/male	Adenocarcinoma	2520 cell / µL
Abughanimeh et al. (our case)	55 years/male	Adenocarcinoma	42090 cell / µL

The production of eosinophils in the bone marrow is regulated by a complex interaction between transcription factors and the presence of eosinophil-promoting cytokines, most importantly IL-5 [2]. In some occasions, eosinophils can be produced outside the bone marrow, similar to allergic inflammation, where it is mediated by IL-5 secreted by the lymphoid cells in the affected tissue [2].  

Several theories have tried to explain the mechanism of eosinophilia in malignancy; some have suggested an extensive dissemination of the cancer in the bone marrow, local stimulation of the adjacent tissue to the tumor, or tumor necrosis as contributing factors [8]. The most acceptable explanation is that the bone marrow, stimulated by release factors such as IL-5, stimulates eosinophil production [7-8]. This was supported by a study by Pandit et al. [9] where they followed the IL-5 levels in a patient with stage IIB lung large cell carcinoma, who had an AEC close to 45000 cells/µL on admission. His AEC and IL-5 levels remained high until the patient had surgical removal of the lung mass, which resulted in a drop of the IL-5 and AEC levels.

Other cytokines can also regulate eosinophil production, such as IL-2, IL-3, and granulocyte-macrophage colony stimulating factor (GM-CSF). Sawyers et al. [10] evaluated two patients with lung cancer and eosinophilia, who had metastasis to the pleura. Upon evaluation of their pleural fluid, both patients were found to have GM-CSF in the fluid. When compared to 11 patients who had pleural effusion without leukocytosis, only one of them was found to have detected GM-CSF. This lead to the conclusion that GM-CSF plays a role in the presence of eosinophilia in malignancies. Another study by Macdonald et al. [15] showed that the use of IL-2 in the treatment of AML patients was associated with an increase in AEC. This was attributed to the IL-2 stimulatory effect on T-cells, which lead to the release of IL-5.

The presentation of paraneoplastic eosinophilia can range from asymptomatic to life-threatening complications such as thromboembolism, especially in cases of hypereosinophilic syndrome (HES) [2]. HES is defined as an AEC greater than 1500 cells/µL for six months and is associated with secondary end-stage organ damage [16]. HES can present with nonspecific symptoms such as cough, fatigue, and night sweats. However, in advanced disease, it can present with symptoms related directly to organ damage, such as skin rash (if it affects the skin), mural thrombus or endocardial fibrosis (if the heart is affected), neuropathy (if the nervous system is affected), or thromboembolic complications (if the blood vessels are affected) [16].

There is no standard treat­ment for paraneoplastic eosinophilia. Unlike primary eosinophilia, which responds to steroids, paraneoplastic eosinophilia can be resistant. Sometimes a combination of steroids and hydroxyurea can increase the response rate [6]. However, the cornerstone of management includes treating the malignancy by either chemotherapy or surgery [4,9]. It is worth mentioning that the presence of paraneoplastic eosinophilia is associated with a poor prognosis and a more aggressive tumor [4].

## Conclusions

Paraneoplastic eosinophilia secondary to lung cancer is rare and usually associated with poor prognosis. There is no specific treatment other than treating the underlying malignancy. Since this condition is rare, workup to exclude a secondary hematologic malignancy or bone marrow disease is needed.
